# Non-union of Conservatively Managed Radial Neck Fractures in Adults: A Systematic Review

**DOI:** 10.7759/cureus.31957

**Published:** 2022-11-28

**Authors:** Alexander Wilton, Hasitha Pananwala

**Affiliations:** 1 Orthopaedics, Northern Sydney Local Health District, Sydney, AUS; 2 Orthopaedics, Ryde Hospital, Sydney, AUS

**Keywords:** nonunion fracture, radius, non-union, fracture, radial neck

## Abstract

Non-union of radial neck fractures in adults is rare. This review aims to identify factors contributing to the non-union of undisplaced radial neck fractures and assess treatment options and outcomes.

Systematic searches of English articles in PubMed, Embase, Ovid Medline, Cochrane Central Register of Controlled Trials, and Cochrane Database of Systematic Reviews were undertaken in September 2021 according to the PRISMA guidelines. The search terms were (fracture) AND (radial neck) AND (non-union OR non-union). Eligible studies reported adults who experienced undisplaced radial neck fractures that went on to non-union without prior surgical intervention.

Fifteen case reports/series were included involving 29 non-unions in 27 patients. The largest study included eight patients. There were 11 males (38%) and 18 females (62%). The average age at the time of the presentation was 55 (range: 29-73). In 13 cases, comorbidities were commented on, including association with smoking in 4 (30%), diabetes in 3 (23%), and excessive alcohol in 5 (38%). The average time from injury to a diagnosis of non-union was 6.7 (range: 2-24) months. The average time of follow-up was 28.6 (range:6-84) months. Eight minimally symptomatic or asymptomatic non-unions were managed conservatively without complication. Seventeen symptomatic non-unions were managed operatively. Treatments included open fixation (1), open fixation with bone grafting (1), bone grafting alone (2), arthroplasty (2), radial head resection (2), and unknown surgery (7). Patients managed operatively achieved full or near-full, asymptomatic range of motion at an average of 5.4 (3-12) months postoperatively.

Non-union is a rare complication of an adult radial neck fracture, and risk factors may include female gender, smoking, diabetes, and chronic alcohol. Persistence with non-operative management is encouraged as it can resolve symptoms with or without a radiographic union. Operative options range from bone grafting +/- fixation to arthroplasty. On average, the time from injury to the decision made to operate is 6.5 (3-12) months. A comfortable, functional range of motion is possible with all treatment strategies.

## Introduction and background

Minimally displaced radial neck fractures are often the result of low-energy trauma, with an incidence of 25 to 30 per 100,000 adults [1-3]. When isolated, these fractures are nearly always impacted with intact periosteum and are unlikely to displace [4]. The prognosis of these injuries is usually satisfactory, with non-operative management and early active range of motion therapy yielding a return to normal function in most patients [5].

This is in contrast to displaced radial head and neck fractures which can form a more severe class of injury and may be associated with elbow dislocation, ligamentous instability, proximal ulna fracture (Monteggia-type injury), or interosseous membrane injury (Essex-Lopresti lesion) with generally poorer long-term outcomes. As a result, experts emphasize vigilance in recognizing these more complex pathologies, which possess a less favorable prognosis [1,2,6,7].

When these complex patterns are excluded, complications related to undisplaced radial neck fractures are rare, and outcomes are usually favorable [8]. Elbow stiffness may persist, for which aggressive physical therapy is usually successful [9,10]. Symptomatic radiocapitellar arthrosis due to undisplaced radial neck fracture is rare [8,11]. Symptomatic non-union of these fractures is a rare occurrence. This systematic review aims to identify the factors that might contribute to adult radial neck non-union in the setting of a minimally displaced radial neck fracture, the natural history of this rare problem, and various treatment strategies available to the patient and orthopaedist.

## Review

Methods

Data Sources

This systematic review was conducted following the preferred reporting items for systematic reviews and meta-analyses (PRISMA). The two authors reviewed the literature independently to identify relevant articles, including PubMed, EMBASE, MEDLINE, and Cochrane databases. Searches were carried out in September 2021. A combination of the following search terms was used: "radial neck", "fracture", "non-union", "non-union" and "delayed union". Reference sections of relevant studies were examined for further additional relevant studies.

Study Selection

Studies were included if they presented characteristics or management of radial neck non-unions in adults without prior elbow surgery. Patients were excluded if their initial injury was Mason grade 2 or 3, associated with the elbow dislocation, or other injuries around the elbow (i.e., Monteggia or Essex-Lopresti type injuries). Both symptomatic and asymptomatic non-unions were included, and studies that reported non-union following fixation or did not separate non-unions from malunions were excluded.

Data extraction

Data were independently recorded in pre-designed spreadsheets. The title, author, year, and level of evidence were recorded for each study. Each patient diagnosed with non-union and delayed union was recorded. Patient demographics collected included sex, age, time from injury to a diagnosis of non-union, and risk factors predisposing to non-union were recorded. Pre-treatment non-union variables included mechanism of injury, initial treatment prior to non-union, whether the non-union was symptomatic, range of motion (ROM), and the primary reason given for intervention were recorded. Post-treatment variables included type of intervention (physiotherapy, shock wave therapy, immobilization, fixation, excision or arthroplasty), bone grafting used, complications, time to radiographic union, time to asymptomatic, post-treatment ROM, post-treatment symptoms and patient-reported outcome measures if recorded. Radiographs included within the text were reviewed to confirm the diagnosis and validity of the inclusion criteria.

Data Synthesis

The authors reached a consensus on articles and data included in this study (AW and HP). Statistical analyses were performed using Microsoft Excel (Redmond, WA: Microsoft Corporation).

**Figure 1:**
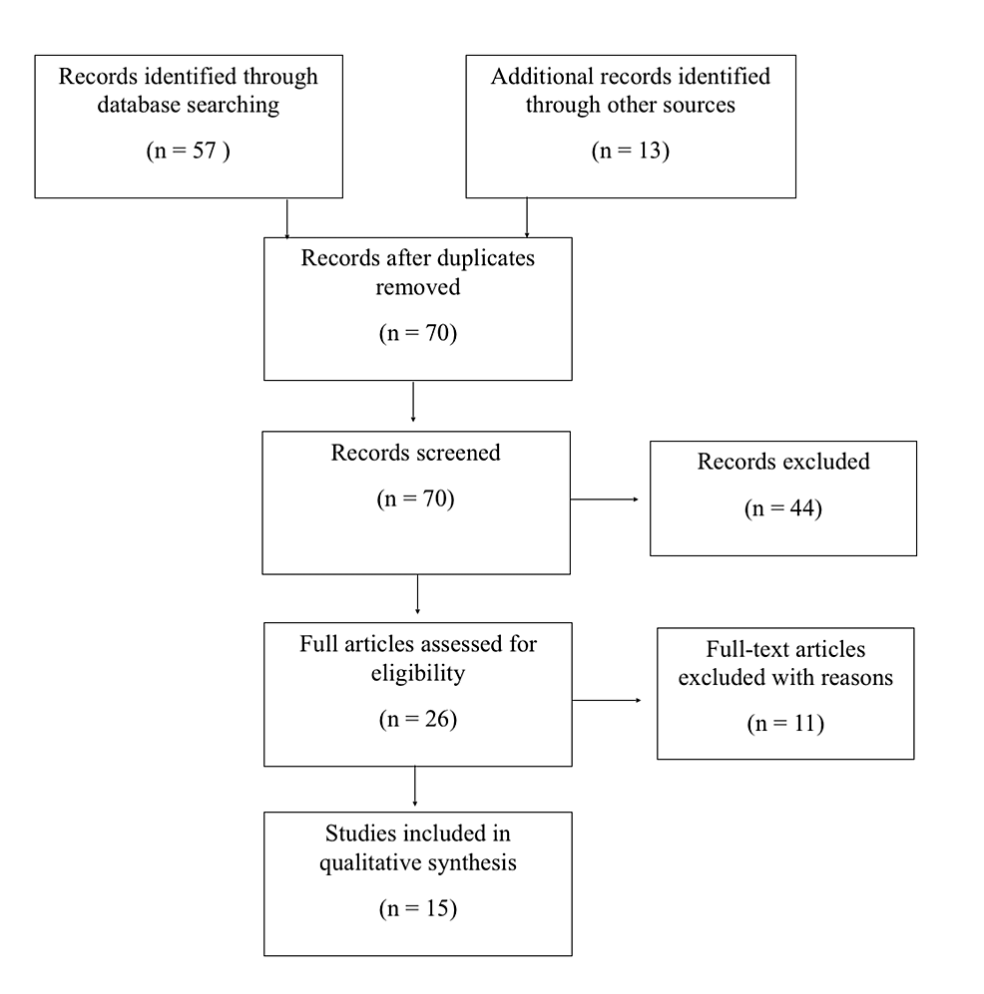
Flowchart of studies included in the review

Fifty-seven studies were obtained after the initial screening, and 13 were identified through other sources. After removing duplicates and applying inclusion and exclusion criteria, 15 studies were included. The details of this process are visualized in the flowchart figure 1. A description of each included study is shown in table 1. Of those studies included, 1 was a retrospective cohort study, 2 were retrospective case series, and the remaining 12 were individual case reports.

Results

Fifty-seven studies were obtained after the initial screening, and 13 were identified through other sources. After removing duplicates and applying inclusion and exclusion criteria, 15 studies were included. The details of this process are visualized in the flowchart in figure 1. A description of each included study is shown in table 1. Of those studies included, 1 was a retrospective cohort study, 2 were retrospective case series, and the remaining 12 were individual case reports.

**Table 1:** Summary of articles included in the review ETOH = Chronic Alcohol Use, DM = Diabetes Mellitus, L = Left, R = Right, PIN = Posterior interosseous nerve

Author	Case no.	Gender	Age	Side	Comorbidities	Time of diagnosis of delayed/non-union and intervention (months)-	Management	Follow-up (months)	Symptoms, at the last follow up	Xray at follow up
Babst et al. [12]	1	M	43	R	-	6	2 lag screws	12	Minor	Union
	2	F	36	L	-	3	Plate	Unknown	No	Union
Cha et al. [13]	3	F	54	L	-	12	Iliac crest graft + K wire	24	No	Union
Cobb et al. [14]	4	F	48	L	ETOH	3	Conservative	48	No	Nonunion
	5	F	64	R	-	5	Cast and bone stimulator	19	No	Union
	6	F	73	L	-	3	Conservative	84	No	Nonunion
	7	F	69	L	-	4	Conservative	16	No	Union
Coury et al. [15]	8	M	66	R	-	6	Radial Head Ressection	24	No	-
Cusano et al. [16]	9	F	70	L	-	4	Arthroplasty	6	No	-
Delattre et al. [17]	10	F	67	L	-	6	Arthroplasty	84	No	-
Faber et al. [18]	11	F	67	R	-	24	Conservative	Unknown	Minor	-
Faraj et al. [19]	12	F	69	R	-	6	Radial Head Resection	-	No	-
	13	F	47	L	Smoker, ETOH	3	Conservative	-	-	-
Golinvaux et al. [20]	14	F	58	R	Smoker	-	Unknown	-	-	-
	15	M	55	R	DM, ETOH, obese	-	Unknown	-	-	-
	16	F	62	R	DM, obese	-	Unknown	-	-	-
	17	F	58	R		-	Unknown	-	-	-
	18	M	35	R	ETOH	-	Unknown	-	-	-
	19	M	47	Bilateral	Smoking, ETOH, cirrhosis, Hep C	-	Unknown	-	-	-
	20	M	54	L		-	Unknown	-	-	-
	21	M	64	R	Smoker, DM	-	Unknown	-	-	-
Karpinski et al. [21]	22	F	59	R		9	Conservative	15	No	Nonunion
Middleton et al. [22]	23	M	29	Bilateral		5, 8	Iliac Crest Graft	24	No	Union
Nakanishi et al. [23]	24	F	73	L		12	Reverse vascularised humeral graft and plate (for PIN palsy related to nonunion)	2	No	Union
Ozcan et al. [24]	25	F	73	L		8	Conservative	24	No	Nonunion
Pace et al. [25]	26	M	38	R		2	Cast 2 weeks	14	No	Nonunion
Salai et al. [26]	27	F	55	R	Rheumatoid	6	Conservative	12	Minor	Nonunion

Pre-intervention Characteristics

**Figure 2:**
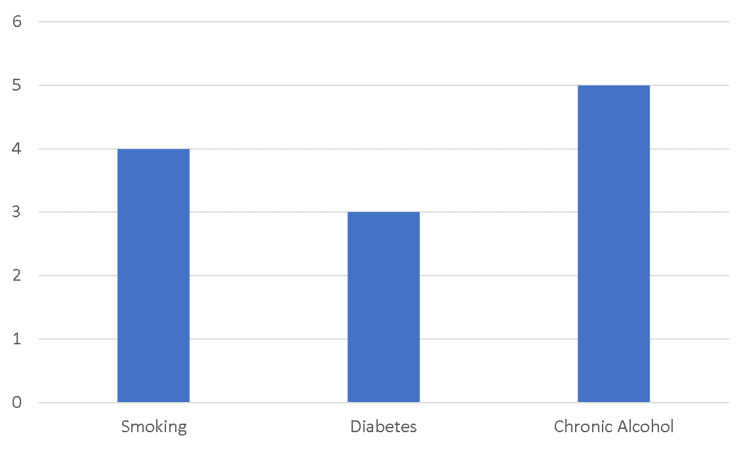
Summary of patient characteristics and follow-up times in case reports and case series. y-axis = number of cases

The 15 studies identified 29 non-unions in 27 patients (table 2). Two patients experienced bilateral non-unions. There were 11 males (38%) and 18 females (62%). The average age at the presentation was 55 (29-73) years old. In 13 cases, risk factors for non-union were commented on. Non-union was associated with smoking in 4 (30%), diabetes in 3 (23%), and excessive alcohol in 5 (38%) cases (figure 2). All non-unions were atrophic. In 19 cases, initial treatment of the fracture was commented on. Initial treatments prior to non-union consisted of immediate physiotherapy with early range of motion in 11 (58%) cases, brief immobilization < 3 weeks, then physiotherapy in 3 (16%), and plaster immobilization for 4 weeks in 1 (5%) case. In 4 (21%) cases, the initial injury was classed as "neglected" as it was not diagnosed or managed at the time of injury. The average time from injury to a diagnosis of non-union was 6.7 (2-24) months. The average time of follow-up was 28.6 (6-84) months. Of the 26 non-unions where pre-intervention symptoms were discussed, 17 (65%) had persistent elbow pain, and 9 (35%) were pain-free.

**Table 2:** Summary of patient characteristics and follow-up times in case reports and case series. n = number

Studies (n)	15
Patients (n)	29
Radial neck nonunions (n)	27
Male (n, %)	11 (38%)
Age at presentation (mean years, range)	55 (29-73)
Time from injury to the diagnosis of non-union (months, range)	6.7 (2-24)
Follow-up (mean months, range)	5.4 (3-12)

Treatment and Outcomes

The management (operative or non-operative) was discussed in 20 cases.

Non-operative: Eight minimally symptomatic or asymptomatic non-unions in 8 patients were treated conservatively with universally excellent outcomes. One patient with 'non-union' diagnosed at five months achieved radiographic union at eight months with electromagnetic stimulation alone. Two patients with painful non-union at presentation were managed non-operatively and became symptom-free at the final follow-up. All patients managed non-operatively were asymptomatic or minimally symptomatic at the final follow-up. Non-operative treatment options included brief elbow cast or sling immobilization (<4 weeks) and early active range of motion physiotherapy.

Operative: Seventeen non-unions in 16 patients were treated operatively. The indication for surgery was 'persistent pain' in all but 1 case (94%), where a patient underwent decompression and arthroplasty for posterior interosseous nerve palsy related to a joint effusion adjacent to the non-union. For all patients undergoing operative intervention, the average time from injury to the decision made to intervene was 6.5 (3-12) months. Treatments included open fixation without bone grafting (1), fixation with bone grafting (15), bone grafting alone (2), arthroplasty (2), resection (2), and unknown surgery (7). All patients with fixation or bone grafting achieved radiographic union at 4 months (2.5 - 8). These included 4 non-unions managed with iliac crest graft and 1 with reverse humeral vascularised graft. All patients managed operatively achieved full or near-full, asymptomatic range of motion at an average of 5.4 (3-12) months postoperatively. Excellent Mayo Elbow Performance Scores were recorded in 3 patients at the final follow-up, averaging 95 (85-100).

Complications: No patients experienced major complications. 2 patients who underwent plate fixation underwent planned removal of hardware after the bony union. One patient managed with a reverse vascularised humeral bone graft experienced minor numbness in the distribution of the posterior cutaneous nerve of the forearm.

Discussion

Radial head and neck fractures are common, with Mason-Hotchkiss type-1 fractures making up the majority of injuries (64% - 82%) [1,2,27]. Isolated radial neck fractures are included within this 'type-1' category and are less common than radial head fractures, generally resulting from lower energy trauma [1]. Undisplaced radial neck fractures are generally managed non-operatively with excellent outcomes [9,28]. The exact non-operative protocol remains controversial and is reflected in our study's variety of immobilization and rehabilitation strategies. One recent randomized trial suggests that formal physiotherapy is unnecessary and not cost-effective [29]. As a result of such excellent outcomes, radiographic investigation of these injuries is often limited to the first few weeks after trauma. It has therefore been hypothesized that the incidence of asymptomatic, undiagnosed non-union of these injuries may well be under-reported [30].

Non-union of the adult radial neck in the context of any elbow injury is rare. Arner et al. retrospectively examined records of 1700 fractures of the head and necks of radii without a single incidence of non-union [31]. Thomas first mentioned the phenomenon of radial neck fracture non-union in 1907 [32]. The first well-documented case was by Middleton at Royal North Shore Hospital in Sydney, Australia, in 1976, where a 29-year-old, very active farmer experienced bilateral non-unions at 5 and 8 months, respectively, both managed successfully with bone grafting [22]. In Golinvaux et al. contemporary cohort of 472 consecutive minimally displaced radial head fractures treated non-surgically over 15 years, only 8 non-unions were found (1.7%) [20]. They commented that their retrospective study design introduced selection bias which may under-report the pathology's true incidence.

The cause of non-union of the radial neck in adults remains unclear. The incidence of non-union of the radial head and neck should be high, as the blood supply to the intracapsular head is arguably tenuous, with limited soft tissue attachments analogous to the head of the femur [33]. The radial neck has been shown in anatomic studies to be encircled by a pericervical ring that obtains blood supply from the recurrent radial artery and interosseous artery [34]. This periarticular supply may offset the apparent risk for devascularization and keep the risk of non-union relatively low when fractures are undisplaced [21]. The rare cases of non-union in undisplaced injuries may result from excessive motion at the fracture site rather than vascular injury [18,24,26]. We have seen that biological factors such as alcohol, smoking, and diabetes may also contribute.

**Figure 3:**
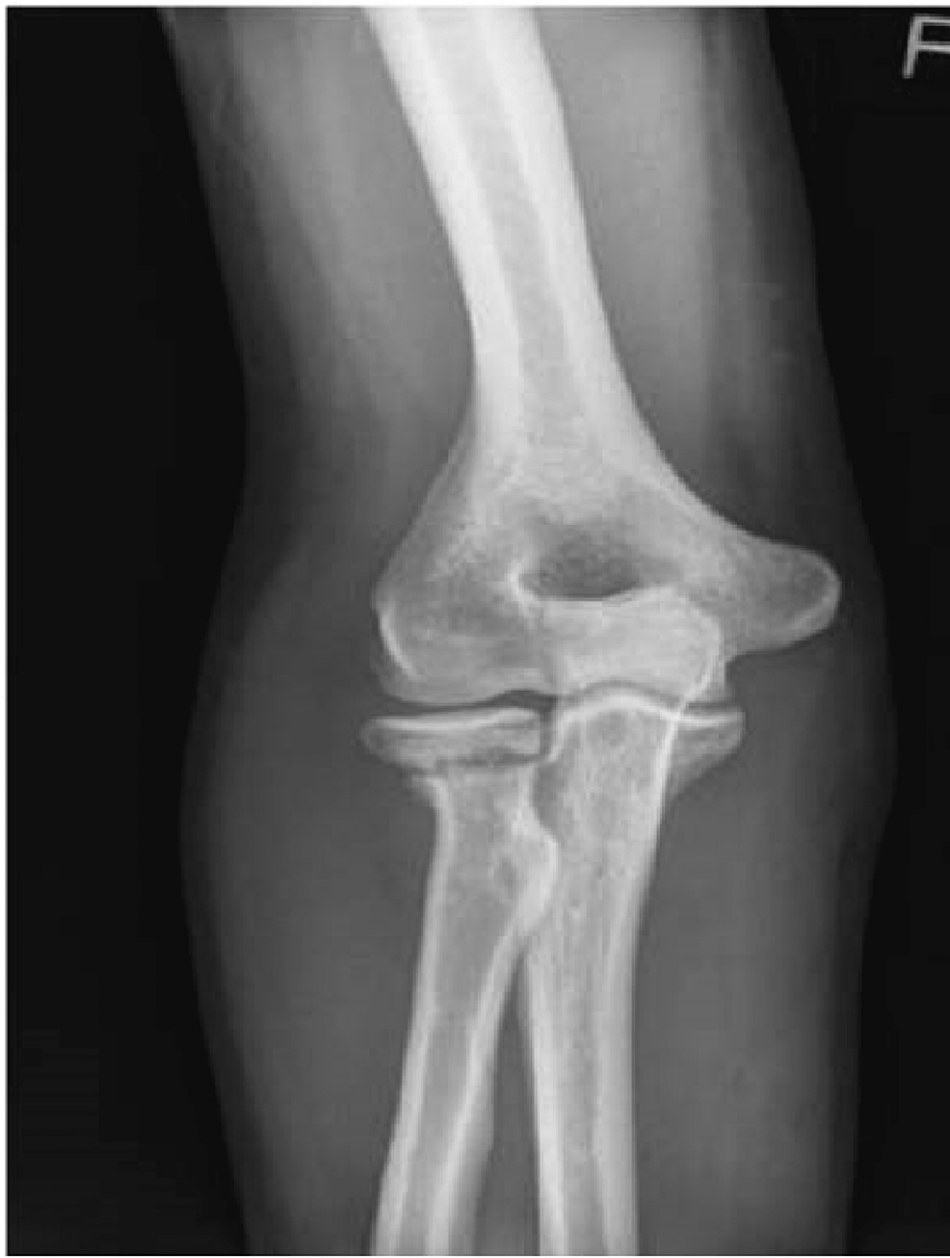
Xray demonstrating an example of radial neck nonunion Reproduced from Pace et al. [23] with permission.

This study illustrates that the management of radial neck non-union should depend on the clinical presentation and not radiographic findings. The major symptom at presentation was pain with activity; however, some non-union symptoms were so mild and acceptable to the patient that no intervention was made [14,18,24-26]. There is a lack of consensus on the timeframe for diagnosis of radial neck non-union. Therefore, deciding when to pivot management to an operative intervention is difficult. Delaying surgery as long as possible seems reasonable, with some cases demonstrating that painful non-union can become painless without operative intervention [24,25]. The imaging of one such case is presented in figure 3. Physiotherapy was utilized in these cases to achieve an early range of motion, and cases included in this study abandoned non-operative management at an average of 6.5 months after injury. The decision for operative intervention was for persistent pain in 94% of cases, with the patient's functional goals cited as a significant contributing factor.

Bone preserving operations such as fixation with screws or plates, with or without bone grafting, were performed in younger patients with an average age of 44. All achieved union in a reasonable time. By contrast, radial head resection (average age 67.5) or arthroplasty (average age 68.5) was generally reserved for older patients with less functional demands. This generally reflects the treatment algorithm for an acute radial head injury. Radial head resection should be considered cautiously as there is a risk of persistent instability and proximal radial migration if ligamentous or interosseous membrane injury is not recognized [35].

This study is limited by its small sample size and inclusion of only case reports and case series. A reliance on case reports is likely to introduce selection bias for cases managed operatively or with positive outcomes. As others have suggested, large prospective series with radiographic follow-up of radial neck fractures are required to understand better the true incidence and natural history of this rare pathology [30,36]. Another major limitation involves variability in the definition of time to non-union between studies. The United States Food and Drug Administration (FDA) defines non-union as a fracture that persists for a minimum of 9 months without signs of healing for three months [37]. Studies included in this review often used 'delayed' and 'non-union' interchangeably, with significant variation in timeframes for diagnosis (see table 1).

## Conclusions

Non-union is a rare complication of an adult radial neck fracture. Risk factors may include female gender, smoking, diabetes, and chronic alcohol. Treatment must be tailored to the patient's functional goals and ranges from physical therapy and bone grafting +/- fixation to arthroplasty. In the literature, the average time from injury to the decision to operate was 6.5 (3-12) months. A comfortable, functional range of motion is possible with all treatment strategies. Persistence with non-operative management is encouraged as symptoms can be resolved with or without a radiographic union.
